# Development and Feasibility of a Regulated, Supramaximal High-Intensity Training Program Adapted for Older Individuals

**DOI:** 10.3389/fphys.2019.00590

**Published:** 2019-05-21

**Authors:** Mattias Hedlund, Nina Lindelöf, Bengt Johansson, Carl-Johan Boraxbekk, Erik Rosendahl

**Affiliations:** ^1^Department of Community Medicine and Rehabilitation, Physiotherapy, Umeå University, Umeå, Sweden; ^2^Department of Public Health and Clinical Medicine, Umeå University, Umeå, Sweden; ^3^Centre for Demographic and Aging Research (CEDAR), Umeå University, Umeå, Sweden; ^4^Danish Research Center for Magnetic Resonance, Center for Functional and Diagnostic Imaging and Research, Copenhagen University Hospital, Hvidovre, Denmark

**Keywords:** sprint interval training, high-intensity interval training, affective state, perceived exertion, training intensity, aging

## Abstract

**Background:** High-intensity training (HIT) with extremely short intervals (designated here as supramaximal HIT) is a time-efficient training method for health and performance. However, a protocol for regulation and control of intensity is missing, impeding implementation in various groups, such as older individuals.

**Methods:** This study presents the development and characteristics of a novel training protocol with regulated and controlled supramaximal intervals adapted for older people. Using both quantitative and qualitative analyses, we explored the feasibility of the program, performed in a group training setting, with physically active older individuals (aged 65–75, *n* = 7; five women). The developed supramaximal HIT program consisted of 10 × 6 s cycle sprint intervals with ∼1 min of active recovery with the following key characteristics: (1) an individual target power output was reached and maintained during all intervals and regulated and expressed as the percentage of the estimated maximum mean power output for the duration of the interval (i.e., 6 s); (2) pedaling cadence was standardized for all participants, while resistance was individualized; and (3) the protocol enabled controlled and systematic adjustments of training intensity following standardized escalation criteria.

**Aim:** Our aim was to test the feasibility of a novel training regimen with regulated and controlled supramaximal HIT, adapted for older people. The feasibility criteria for the program were to support participants in reaching a supramaximal intensity (i.e., power output > 100% of estimated VO_2_ max), avoid inducing a negative affective response, and have participants perceive it as feasible and acceptable.

**Results:** All feasibility criteria were met. The standardized escalation procedure provided safe escalation of training load up to a supramaximal intensity (around three times the power output at estimated VO_2_ max). The participants never reported negative affective responses, and they perceived the program as fun and feasible.

**Conclusion:** This novel program offers a usable methodology for further studies on supramaximal HIT among older individuals with different levels of physical capacity. Future research should explore the effects of the program in various populations of older people and their experiences and long-term adherence compared with other forms of training.

## Introduction

Despite the importance of physical activity and exercise for good health and well-being (Cicioni-Kolsky et al., 2013), less than one third of people over age 75 years reach the World Health Organization recommendation of at least 150 min of physical activity at moderate intensity per week. With the growing aging population, society faces major health-related challenges, and finding effective forms of exercise is crucial (Paterson et al., 2007). Among older individuals, studies suggest that lack of time is an important barrier to engaging in regular physical exercise (Trost et al., 2002; Cohen-Mansfield et al., 2003).

In recent years, the concept of extremely short-duration high-intensity interval training (HIT) has been developed as a time-efficient method to improve health and performance, at least for young and middle-aged people (Babraj et al., 2009; Jakeman et al., 2012; Metcalfe et al., 2012; Adamson et al., 2014a). The broad term “HIT” includes a variety of options, with workloads both below (submaximal) and over (supramaximal) the power output corresponding to 100% of maximum oxygen uptake (VO_2_ max). Here, we are particularly interested in a specific form of HIT with extremely short duration at a supramaximal intensity, which we refer to as “supramaximal HIT” (Laursen and Jenkins, 2002). Of interest, supramaximal HIT has a broad effect on many physiological systems and seems to induce at least comparable effects to endurance training on cardio-metabolic health, but in a fraction of the time (Hazell et al., 2010; Iellamo et al., 2014; Gillen et al., 2016; Ruffino et al., 2017). A few attempts have been made to evaluate the concept of extremely short-duration supramaximal HIT intervals for older people. Adamson and co-workers (Adamson et al., 2014a, b; Adamson et al., 2018) presented promising findings of effects on physical function, cardiovascular health, metabolic health, and self-reported health. However, to our awareness, no method are available for regulating and controlling training intensity in supramaximal HIT protocols in a group training setting, impeding implementation of supramaximal HIT among the general public (Hardcastle et al., 2014; Biddle and Batterham, 2015; Del Vecchio et al., 2015; Follador et al., 2018).

In most supramaximal HIT programs examined, the instruction is to put in maximal effort against a braking force, set in proportion to the person’s body weight, with no restriction in pedaling cadence (Burgomaster et al., 2005; Gillen et al., 2016). The idea is to put in maximum effort throughout every interval during each training session. Thus, when exercising at maximum capability, the development of fatigue causes power output to gradually decrease from interval to interval (Ohya et al., 2013). Of note, even if the perception of exertion and discomfort during some supramaximal HIT programs reaches intolerable levels (Follador et al., 2018), studies among young and middle-age participants have revealed that supramaximal HIT might be experienced as equally or more pleasant than traditional aerobic endurance training at a moderate intensity (Bartlett et al., 2011; Jung et al., 2014; Heisz et al., 2016; Kong et al., 2016; Thum et al., 2017). Yet, the opposite has also been reported (Oliveira et al., 2013; Hardcastle et al., 2014). A general problem with existing training protocols that limits their broad applicability is the lack of guidance for determining appropriate training intensity. Inexperienced non-athletes and older individuals appear to lack the experience needed for proper pacing at supramaximal intensities, leading to difficulties in finding an appropriate target intensity (Hebisz et al., 2016). In addition, even if the instruction is to exercise at a maximum effort in all-out sprints, pacing is used to end up with a sustainable and acceptable training intensity (Billaut et al., 2011; Hureau et al., 2014). Hence, without proper guidance, the risk is significant for both under- and over-shooting exercise intensity (Hebisz et al., 2016), compromising training efficiency and increasing the risk of dropout from a negative affect (Perri et al., 2002; Stathi et al., 2010; Follador et al., 2018). If a training program, e.g., supramaximal HIT, is to have a broad, persistent, and profound effect generally, the affective response during exercise is important (Kiviniemi et al., 2007; Williams, 2008; Lee et al., 2016; Cox et al., 2018). This aim is sometimes difficult to achieve, and clinical trials show that the dropout rate is approximately 50% in the first few months after the start of any exercise intervention (Dishman and Buckworth, 1996). Discomfort and negative affective state, e.g., because of high intensity during exercise, have been identified as important reasons for dropout (Perri et al., 2002; Stathi et al., 2010). In this context, a tailored exercise program with controlled supramaximal HIT is clearly warranted (Vollaard et al., 2017) because many previous protocols have been considered so demanding that they cannot be recommended to the general older population (Hardcastle et al., 2014; Follador et al., 2018). In addition, as Shepherd co-workers have noted (Shepherd et al., 2015), most of the studies with supramaximal HIT have been performed with small samples. They have involved one participant at a time in a laboratory setting where the test leader adjusts the training load. For these reasons, these studies are not applicable for a real-world context, which often includes group training.

In a series of pre-study pilot experiments, we developed an adapted training program with regulated and controlled supramaximal HIT intervals, suitable for older and untrained individuals. The intention was to construct a program that could become broadly applicable and be conducted on a group basis in an ordinary gym facility.

### Aim

The aim of this study was to test the feasibility of a novel training regimen with regulated and controlled supramaximal HIT intervals adapted for older people. For the program to be considered feasible, results needed to demonstrate that the training program:

- supports participants in reaching supramaximal intensity;
- does not induce negative affective responses; and
- is perceived by participants as feasible and acceptable.

## Materials and Methods

### Development of the Training Program

We named the program “RAISE-HIT,” an acronym for *Regulated, Adapted Interval Sprints with a standardized Escalation procedure*. The final program was inspired by and adapted from previously developed supramaximal HIT programs (see, e.g., Jakeman et al., 2012; MacLean et al., 2018). The program consisted of 10 × 6 s of cycling intervals, interspersed with ∼1 min of recovery. Important key characteristics of the program were as follows:

1) The participants had an individual target power output (TPO) to reach and maintain at an even pace during the interval sprints. TPO was expressed as the percentage of estimated maximum mean power output (MPO) for the duration of the interval and was the same for all intervals throughout a training session.
2) TPO was reached with a standard pedaling cadence, which was kept equal for all participants, regardless of individual capability (85 rpm during the 6-second intervals and 60 rpm during warm-up and recovery), while the brake level was set in relation to individual capability.
3) The protocol enabled a systematic escalation of training intensity, reducing the risk of discomfort and adverse events from overshooting the training intensity, as well as suboptimal training stress from undershooting of training intensity. Each escalation step was equally large in absolute terms (watts) during the training period.

The program was developed through a series of pilot studies and pre-study experimental procedures. Description of the setup and some underlying considerations are outlined in Appendix 1. During the pilot experiments, we observed that the standardized training protocol, along with a few simplifying utilities (see Appendix 2), provided a sufficiently valid control of the power output during the intervals. Even complete novices of watt-based training learn on their own to adjust the training load in a few seconds and get very close to the stipulated TPO (±5%) during the first training session.

### Intervention

The intervention was a 6-week cycle training class in a gym setting equipped with training bikes with constant monitoring (but not sampling) of, e.g., watts, pedaling cadence, and heart rate (Tomahawk IC7, Indoor Cycling Group, Nürnberg, Germany). A prolonged recovery between sessions has been suggested for older people (Herbert et al., 2015). Training frequency was therefore set to two sessions per week with at least 72 h between training sessions. Before the intervention, the participants performed two pre-tests to determine training intensity.

### Participants

Study participants (*n* = 8; 5 women) were recruited from existing senior training groups at the training facility. Inclusion criteria were between 65 and 75 years of age, regularly physically active (i.e., met WHO criteria for physical activity and exercised at an intensity where they experienced increased breathing at least twice a week), and no neurological or other medical conditions. One of the participants was former elite athlete, while most of the participants had quite recently started with regular exercise training. Prior to inclusion in the study, all participants underwent a medical examination by an experienced cardiologist to identify and exclude individuals with any medical condition for which moderate-to-high intensity exercise was contraindicated. The participants were not to have had any experience of watts-based cycle training. To avoid an increase in total exercise volume, participants were instructed to replace two of their ordinary training sessions with supramaximal HIT every week. The participants gave informed consent after receiving verbal and written information about the study. The study was approved by the Regional Ethics Review Board in Umeå (DNR: 2016-279-31M, DNR 2016-440-32M).

One of the eight originally included participants was excluded after nocturnal cardiac arrhythmia was noted on analysis of heart rate variability data collected during baseline testing before the intervention (heart rate variability data were not analyzed in this study). After discussion with a cardiologist, we excluded the participant from the intervention with a referral to cardiologic consultation. In total, seven participants (five women) completed the training intervention (Table 1).

**TABLE 1:** Baseline characteristics.

	Median	Min–max	Mean (±SD)
Age, years	67	65–72	68.1 ± 3.2
APMHR, bpm	161	157–163	160.0 ± 2.3
Weight, kg	72	58–103	74 ± 14
VO_2_ max, l/min	1.6	1.3–3.5	1.9 ± 0.7
VO_2_ max l/min (percent of predicted; Schneider, 2013)	107	92–163	107 ± 33
MAP, watts	108	78–146	127 ± 54
Maximum MPO^6^, watts	341	239–613	115 ± 94
Maximum workload on BCST, watts	184	148–365	223 ± 69

*APMHR = age predicted maximum heart rate (208 - age × 0.7; Tanaka et al., 2001); VO_2_max = estimated maximal oxygen uptake (Pescatello, 2014); MAP = estimated maximum aerobic power output (Tronarp et al., 2018); Maximum MPO^6^ = estimated maximum mean power output for 6 s (Borg, 1982). Maximum workload on BCST = Peak workload on the Borg Cycle Strength Test (Graneheim and Lundman, 2004).*

### Pre-testing

#### Submaximal Estimation of Maximum MPO for Six Seconds

The pre-test included two submaximal tests used to determine the TPO during the different intensity zones in the training program, namely, the submaximal estimation of maximum aerobic power output (MAP) and the submaximal estimation of maximum MPO for 6 s (maximum MPO^6^).

Estimation of MPO^6^ was performed using the Borg Cycle Strength Test (BCST; Borg, 1982). The BCST is a submaximal ramp test for estimation of maximum MPO for a duration of 30 s (MPO^30^) without requiring exercise at maximal effort, as in the Wingate test (Ayalon et al., 1974). The original BCST is described in detail elsewhere (Borg, 1982). Appendix 3 gives a brief description of the functioning of the test including an explanation of some modifications made in this study to suit our population of interest. After establishing maximum MPO^30^, we derived maximum MPO^6^ by multiplying MPO^30^ by 1.4. This step was based on data in the literature regarding the ratio of mean power over 5 s to the mean power over 30 s during a maximum 30-second Wingate test in non–strength-trained older adults (Slade et al., 2002).

#### Submaximal Estimation of Maximum Aerobic Power Output

Estimation of maximum oxygen uptake (VO_2_ max) was done using the Åstrand-Rhyming test. Workload for the test was individualized based on the American College of Sport Medicine’s recommendations (Pescatello, 2014), which resulted in workloads of 50–75 watts and 75–150 watts for women and men, respectively. The participants cycled at a constant pedal cadence (60–70 rpm) for 6 min and rated their perceived exertion according to the Borgs RPE scale (Borg, 1998; MacLean et al., 2018) at the completion of each minute of the test. The submaximal test was performed on an ergometer bike (Monark Exercise AB, Sweden) with speed-independent power control. Heart rate was monitored continuously using a heart rate monitor (Polar V800, Polar Electro Inc., Finland), which wirelessly connected to a computer that logged the heart rate. The estimated VO_2_ max from the test was then used to mathematically derive the MAP. MAP is the power output (in watts) corresponding to the estimated value of VO_2_ max (l/min) by interpolation of the quadratic equation based on Åstrand-Rhyming data: −1.61x^2^ + 83.72x − 23.48, where x represents the estimated VO_2_ max (Åstrand et al., 2003).

### Training Protocol

**FIGURE 1:**
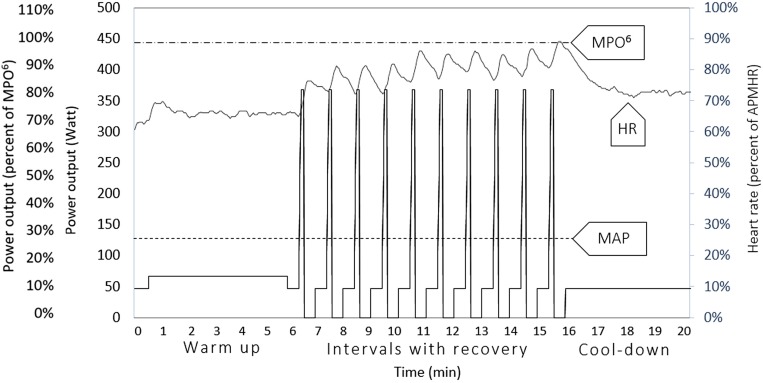
Structure of the supramaximal HIT-program. The left *y*-axis represents power output expressed as both percent of maximum mean power output for a duration of 6 s (*MPO^6^*) and absolute power output (watts). The dotted horizontal line at the top represents the group mean value of MPO^6^, estimated at the pre-test. The lowest dotted horizontal line represents the group mean value of estimated maximal aerobic power output, estimated at the pre-test. That is, the power output corresponding to the estimated VO_2_ max. The solid line represents the group mean value of target power output at the end of the 6-week training period. The overlaid gray line in the figure is a representative graph of the heart rate response from the last session of the 6-week training period for one of the participants, expressed as percent of age predicted maximum heart rate, read on the right *y*-axis.

At the first training session, the group was introduced to the functioning of the training bikes and how to regulate the training load (with cadence and brake level) to reach the stipulated TPO. They were also familiarized with the training program through a slightly modified training session with only five intervals and with doubled rest periods between intervals. From the second session, each training session included 10 intervals of 6 s each. Intervals started every minute. Each training session started with a warm-up (6 min) and ended with a cool-down (5 min). In total, each training session lasted 20 min (Figure 1).

Three TPO workload levels were used in the program and were referred to as intensity zones. Zone 1 was used during recovery and cool-down, Zone 2 was used during warm-up, and Zone 3 was the TPO during the intervals. Zones 1 and 2 were set as a fraction of the person’s estimated MAP at the pre-test, and Zone 3 was set as a fraction of the person’s estimated maximum MPO^6^ at the pre-test (described above). Escalation of exercise intensity during the 6-week training period applied only to Zone 3. Zone 1 and Zone 2 always corresponded to 33 and 50%, respectively, of the individually estimated MAP from the pre-test. At the start of the program, Zone 3 corresponded to 65% of the estimated maximum MPO^6^, whereupon the intensity was escalated according to a standardized escalation procedure. Twenty-five seconds prior to the start of each interval, the brake level was set to Zone 1, followed by a 5-second preparation period to allow time to adjust the brake level up to the current stipulated TPO for Zone 3, increasing the pedaling cadence from 60 rpm up to 85 rpm, and then holding that cadence at an even pace for 6 s. After each interval, the brake level was set to zero for 24 s to optimize recovery and allow for collection of ratings of perception of exertion and affective state.

### Intensity Escalation Procedure

To allow time for learning and to avoid discomfort and adverse events from overshooting the training intensity, the TPO for the intervals (Zone 3) was gradually increased following a standardized escalation scheme. The initial intensity was set to 65% of maximum MPO^6^. Each escalation step was equally large in absolute terms (watts) during the entire training period (i.e., 5% of estimated MPO^6^). Thus, the relative increase in intensity gradually decreased for each step. The instructions before and during the training program included the concept that the training intensity should escalate during the training period to reach an optimal training intensity. Certain criteria were yet to be met to allow the participant to take the next escalation step in the scheme (Table 2). Correspondingly, the intensity was de-escalated in steps of 5% of estimated MPO^6^ if any criteria were met that suggested that the load was too high. The participants were blind to these criteria. The criteria were based on the participant’s (i) ability to produce TPO, (ii) rating of perceived exertion, (iii) assumption about their ability to conduct at least one additional interval at the same power output, and (iv) confidence in their ability to manage the next step in the escalation scheme. The participants maintained the same step for the next session if the escalation or de-escalation criteria were not met.

**TABLE 2:** Escalation and de-escalation criteria as the basis for the decision on intensity for the next training session.

Escalation of TPO if all of the following criteria are met at the 10th interval	De-escalation of TPO if any of the following are met at the 10th interval
– Cadence ≥ 85 rpm during more than half of the duration of the interval	– Cadence < 85 rpm during more than half of the duration of the interval
– RPE score ≤ 16	– RPE score ≥ 18
– An additional interval had been possible	– An additional interval had not been possible
– Ready to escalate load next session	– Wanted to de-escalate load next session

*TPO, target power output.*

### Setting and Equipment

The group training was conducted in a separate room in the gym. Audiovisual supporting information was displayed on a large screen with external speakers. The information was streamed from a mobile phone application with which the training program had been automated (Seconds Interval Timer, Runloop Ltd.). This information included the current interval number, and when to change the intensity zone. To facilitate and speed up regulation of the brake force level, a custom-made cardboard sheet was mounted on the bike (Figures A2, A3 in Appendix 2). In addition, information regarding pedaling cadence and brake force level for each intensity zone was presented on an individual cardboard sheet attached on the bike next to the monitor (Figures A2, A4 in Appendix 2). Two supervisors (physiotherapy students) ran the training program. The supervisors walked around among the participants, enabling direct contact as well as enabling collection of the scores on the subjective rating scales at stipulated time points during the session. The leaders also gave some additional preparatory information and encouragement, e.g., “There are 10 s left until you are supposed to increase load and pedaling cadence,” or “Good work on the last interval, everybody.” However, encouragements were kept modest to avoid overshooting the TPO by pushing the participants to attempt a too-high pedaling cadence. For the same purpose and to avoid distracting stimuli, no music was played during the workout. The gym had written procedures for how to react in the event of acute medical conditions. In addition, a registered physiotherapist was always present during the training sessions.

### Data Collection, Analysis, and Presentation

The test of program feasibility relied on quantitative and qualitative data. The quantitative data concerned training intensity, heart rate response, perceived exertion, and perceived affective state during training and were monitored and collected during each training session. The qualitative data considered participants′ perceptions of the training method and experiences of participating in the exercise intervention.

#### Quantitative Data

We investigated the standardized intensity escalation procedure by analyzing how the escalation procedure was followed and at which training session the participants met one or more escalation criteria (hereafter called the “end of escalation”). Furthermore, the absolute and relative training intensities are presented for the following three time points: (Cicioni-Kolsky et al., 2013) the first complete training session, (Paterson et al., 2007) the session at the end of escalation, and (Cohen-Mansfield et al., 2003) the last training session. The stipulated TPO (watts) was assumed as the value for the absolute training load. The relative training intensity was expressed as the percent of estimated maximum MPO^6^ and the percent of estimated MAP. Heart rate was continuously sampled by means of a Bluetooth chest heart rate monitor (Polar V800, Polar, Kempele, Finland), and the lowest (min) and highest (max) heart rate response for every interval during the sessions was recorded as the percent of age predicted maximum heart rate (APMHR).

The perception of exertion during training was investigated by monitoring the RPE response during each training session. The RPE responses were collected promptly after every second interval for a total of five time points for each training session. The ratings were done after completion of the intervals by the supervisors, but participants were instructed to try to report the score corresponding to their perceived exertion during the very last seconds of the intervals. Because each supervisor had to collect ratings from up to four participants, the whole rating procedure took up to 20–25 s. The lowest and highest rated scores during each session were chosen in the analysis to facilitate understanding of how RPE changed over the training session. The RPE responses are presented for the same three training sessions as for training intensity (see above).

The affective state during the training session was investigated by monitoring the affective state response with ratings on the *Feeling scale* (Hardy and Rejeski, 1989). The Feeling scale is a 10-point bipolar scale and ranges from +5 to −5, with verbal anchors of +5 = very good, +3 = good, +1 = fairly good, 0 = neutral, −1 = fairly bad, −3 = bad, and −5 = very bad. Instructions for this scale were as follows: *While participating in exercise, it is common to experience changes in mood. Some individuals find exercise pleasurable, whereas others find it to be unpleasant. Additionally, feelings may fluctuate across time during a single training session. That is, one might feel good and bad a number of times during exercise. Scientists have developed this scale to measure such responses.* The affective state response was rated at the following four time points during each training session: (1) at rest before warm-up, (2) after the fifth interval, (3) after the tenth interval, and (4) at the end of the cool-down period. Affective state responses are presented for the same three time points as for training intensity and perceived exertion (see above).

In addition, the practicability and acceptance of the program was investigated by analyzing the attendance rate.

#### Qualitative Data

Qualitative data were collected by an interview in one focus group with six participants. The focus group interview was performed directly after the last training session. A semi-structured interview guide was used with questions about experiences about participating, practical issues, how the training affected the participants, and their thoughts about performing HIT among untrained individuals.

#### Data Analysis

The qualitative data resulted from descriptive presentations in the focus group interview. The audio-recorded interview was transcribed, and the transcript was condensed and coded according to qualitative content analysis (Graneheim and Lundman, 2004). The codes were deductively sorted into categories corresponding to the areas of feasibility, i.e., perceptions of the exercise intensity, perceived exertion, and affective state during and after exercise, and if the program, in a broad sense, was experienced as doable and acceptable. The findings are described for each area and illustrated by quotes from the focus group.

The quantitative data were analyzed using IBM SPSS Statistics for Windows, Version 24.0. (Armonk, NY: IBM Corp.) and are presented descriptively with both median (range, min–max) and mean (± SD) values (Table 3).

**TABLE 3:** Training intensity, perception of exertion, and affective state response for three time points during the training period.

*A) Training intensity*									
	First complete session**			End of escalation			Last session		

	Median	Min–max	Mean (±SD)	Median	Min–max	Mean (±SD)	Median	Min–max	Mean (±SD)
***Intensity at Zone 3***									
*Watts*	268	155–420	276 ± 100	339	227–549	366 ± 121	339	239–521	368 ± 118
*Percent MPO^6^*	65	60–65	64 ± 2	90	65–90	87 ± 11	90	65–100	88 ± 12
*Percent of MAP*	227	168–263	219 ± 42	286	223–385	296 ± 69	292	223–385	299 ± 67
Percent of APMHR (min)*	72	65–87	74 ± 8	82	67–97	81 ± 11	78	67–97	79 ± 11
Percent of APMHR (max)*	85	70–99	84 ± 11	89	77–99	91 ± 8	91	74–106	88 ± 11

***B) Perception of exertion***									

**RPE at:**									
*Warm-up*	9.5	9–12	9.8 ± 1.2	9.5	9–11	9.7 ± 0.8	9	8–11	9.3 ± 1.1
*Min during intervals*	11	9–13	11.0 ± 1.5	13	12–14	12.8 ± 0.8	13	9–14	12.3 ± 1.9
*Max during intervals*	14	13–15	14.2 ± 0.8	15.5	15–17	15.8 ± 1.0	14.5	13–17	14.7 ± 1.6
*End of cool-down*	10.5	9–15	11.2 ± 2.4	9	9–11	9.5 ± 1.1	9	8–12	9.3 ± 1.4

***C) Affective state response***									

**Feeling scale at:**									
*Before warm-up*	3	1–5	3.0 ± 1.8	3	1–4	2.7 ± 1.4	3.5	1–4	3.2 ± 1.2
*After 5^th^ interval*	2.5	1–5	2.7 ± 1.4	3	1–4	3.0 ± 1.1	3.5	1–4	3.2 ± 1.2
*After 10^th^ interval*	3	1–4	2.7 ± 1.0	3	1–4	3.0 ± 1.1	4	1–5	3.5 ± 1.4
*End of cool-down*	3.5	1–5	3.3 ± 1.4	3.5	1–5	3.5 ± 1.2	5	1–5	4.0 ± 1.7

MPO^6^, maximum mean power output; MAP, estimated maximum aerobic power output; APMHR, age predicted maximum heart rate (208 − age × 0.7); RPE, rating of perceived exertion. *Percent of APMHR min and max is the lowest and highest heart rate response during intervals, expressed as percentage of APMHR. **First complete session is the first session with 10 completed intervals, i.e., the second training session.

## Results

### Training Intensity

Six of seven participants followed the preplanned escalation scheme. Counted from the first session, the end of escalation was reached at session 6, 7, or 8 (median session 7) for six of the participants. At the session for end of escalation, no participants met the criterion: “Feels ready to increase to the next escalation step next session.” In addition, four of the participants did not meet the criterion of a maximum RPE score ≤ 16. TPO at the end of escalation was in median, and the median TPO was 2.9 times higher than the power output corresponding to their estimated MAP. Median relative intensity at the end of escalation was 90% of maximum MPO^6^. After reaching end of escalation, only one participant could later on escalate TPO further and maintain the new TPO. Therefore, the group mean TPO at the last training session was similar to the TPO at the end of the escalation session. During the training period, two of the participants also met one or more criteria for reduction of training intensity. One participant already met the criterion of cadence ≥ 85 rpm during the majority of the interval at the first session (see below). The other participant met most of the de-escalation criteria (Table 2) at a TPO of 95% of MPO^6^ at session number 11, which was their first attempt at that escalation step.

One participant, for whom MPO^6^ might have been overestimated, already met several de-escalation criteria at the first session. However, after de-escalation of training intensity to 60% of maximal MPO^6^ at the second training session, the participant could complete an entire training session and then could further escalate training intensity up to 65% of maximal MPO^6^ at the third group training session. The participant could thereafter maintain that escalation step during the training period. However, no further increase was possible because not all criteria for escalation were met.

The median minimum interval heart rate response, expressed as percent of APMHR, was 72% (65–87%) at the first session, 82% (67–97%) at the end of escalation, and 78% (67–97%) at the last session. The median maximum interval heart rate response, expressed as percent of APMHR, was 85% (70–99%) at the first session, 89% (77–99%) at the end of escalation, and 91% (74–106%) at the last session.

In the focus group, the participants reported that it was fun and valuable to be challenged in the way they were by the program. They found that this kind of challenge diminishes with age and that both internal and external factors may drive this phenomenon. Participants further described that it was fun to get outside of one’s comfort zone and that they would not have performed this type of training if it were not in this organized form.

I think that this has been really fun. I am used to exercising a lot, but in recent years I have been training within a comfort zone all the time. I have not thought about exhausting myself more than necessary or more than what feels comfortable. So, it has been great fun to really test and exert myself. I don’t think I would have done this unless the training was supervised.

Guidance and support felt nice and were seen as important because it took some time to get to know where the boundary actually was and to learn how “hard” they in fact could exercise. There was also some uncertainty about their limits of effort, which created some concern and negatively affected the experience. They found it interesting to see the heart rate response during the training sessions, even though they did not fully know how to interpret that information. Therefore, they requested more information and education on these topics and considered it especially important if assuming that the training program were to be performed among older untrained individuals.

To get information about what is happening in the body while exercising if you are not used to training yourself … the heart rate starts to shoot up, your breathing is heavier, and you get the soreness of lactic acid in your legs. It is good to know what is happening in the body and that it is not dangerous.

### Perceived Exertion and Affective State

The median (range) RPE response during the warm-up was 9.5 (9–12) at the first session, 9.5 (9–11) at the end of escalation, and 9 (8–11) at the last session. At the end of the cool-down period, the median RPE score was 10.5 (9–15) at the first session, 9 (9–11) at the end of escalation, and 9 (8–12) at the last session. These intensity zones were conducted at the same TPO during the entire training period. During intervals, the intensity escalated during the training period. The lowest (min) RPE score was always rated during one of the first two intervals of the session, and the highest (max) RPE score was always rated during one of the last two intervals of each session. Both min and max RPE scores increased up to the end of escalation session. The group median of max RPE score during intervals was 14 (13–15) during the first complete training session, 15.5 (15–17) at the end of escalation, and 14.5 (13–17) at the last training session.

The affective state response, as measured with the Feeling scale, was found to be rather similar during the training sessions. The scoring was not at any point found to be negative for any participant. During the first training session, the median score on the feeling scale was 3 (1–5) during warm-up, 2.5 (1–4) at the fifth interval, and 3 (1–4) at the tenth interval. At the end of the cool-down period, the affective state was 3.5 (1–5). At the end of the escalation session, the affective state response was similar to the first training session despite a 20–30% higher training load. At the last training session, the median score was 3.5 (1–4) during warm up, 3.5 (1–4) at the fifth interval, and 4 (1–5) at the tenth interval. At end of the cool-down period during the last session, the affective state was found to be 5 (1–5).

According to the focus group, the training did not appear to have caused any pain or discomfort other than the bicycle saddle, which some perceived as uncomfortable. Rather, the participants expressed that the training program was very comfortable and fun to participate in.

“I have not discovered until now how much fun it is to exercise.”

The participants described that they had noticed always feeling more alert and energetic after training sessions compared to before. This experience was described as somewhat surprising because they were used to feeling a bit tired and exhausted after their usual workouts, such as group hydrotherapy exercises.

Even I wondered if I could manage to exercise with high intensity, but I am not at all deadly tired after the exercise session. I feel good.

They also described positive feelings from the training having been conducted as a group class was enjoyable and inspiring. To participate in a program called “HIT training” was emphasized as motivating and creating a sense of pride:

I’m thinking that, YES, I as an older person can exercise with high intensity!

There was a discussion about whether the program would be better with or without music. One opinion was that it is more fun to exercise with music. Others considered it nice without music and that music would be distracting from the concentration the program required.

“*When you are a little older you may not be able to handle that much stimulus*.”

An idea from the participants was that music could be played during warm-up and cool-down because music during a workout can contribute to a positive feeling.

### Practicability and Acceptance

The median attendance rate of the 12 training sessions was 87.5% (75–92%). The focus group did not reveal anything indicating that the program was perceived as undoable or unacceptable. Rather, the opposite was proposed even if potential participants might be untrained individuals. They considered that a short program like this was interesting and comfortable to perform, although “*for untrained persons, a leader is a prerequisite for it to work.*” The relatively low level of unpleasant muscle pain during the short intervals was considered beneficial and appealing to untrained individuals.

The participants some practical problems, especially in the beginning of the exercise period, such as handling and settings on the bike or feeling it a bit tricky to learn how to simultaneously adjust both the brake level and cadence during cycling. This complexity could create a sense of stress during the first training sessions.

“*It was a little stressful at first, when we should adjust and … my vision is also a bit poor.*”

In addition to wanting information about what was happening in the body when conducting this kind of exercise, the participants also expressed a wish to receive more information about, for example, how the bicycle works and the best sitting position on the bike. To complete all intervals without excessive fatigue and because the perception of exertion grew during the training session, they expressed the importance of not being tempted to overshoot the pedaling cadence and power output during the first intervals.

It was difficult to hold the cadence down to 85. Maybe that is my temper. Some energy got lost that way. It is my first time on this kind of bike, and I have to learn how much effort to use.

The program was perceived as a good complement to the participants’ usual training and suitable for combining with another training session in direct connection to the HIT session. Some participants described improvements that they attributed to the HIT training period, such as improved fitness, better mood, and less pain. They emphasized that HIT increased self-confidence about this particular training that could be transferred to other training because they dared to, and were able to, exert themselves in other such situations as well as in everyday life.

Being able to perform an indoor walking session has been a lift for me, and this is a result of this training group… I have gained more self-esteem as well as more self-confidence in the training situation.

## Discussion

The overall finding is that the developed supramaximal HIT program seems feasible, and all criteria for feasibility that were set prior the study were met. The participants reached a high training intensity at a supramaximal level. The standardized escalation scheme seems to be a usable tool to support a controlled and safe escalation in the training load, up to a high intensity, with low risk of sudden overload caused by overshooting. The analysis also revealed that the training program never induced discomfort or negative affective responses and that it was reported to be perceived as doable and acceptable.

One key characteristic of the program is that the intervals were conducted at a regulated and controlled intensity, i.e., at a stipulated TPO during all the intervals, instead of exercise at subjective “maximum effort.” Most non-athletic individuals find it difficult to know how hard they can work during a supramaximal sprint (Hebisz et al., 2016). This is in line with our clinical experiences and experiences from research on physical performance and exercise training among older individuals (Rosendahl et al., 2006; Hedlund et al., 2012; Lindelöf et al., 2012; Jonasson et al., 2017). An interesting but complicating issue is that supramaximal HIT with extremely short intervals differs fundamentally from other forms of endurance training. The absolute workload that provides stimuli for adaptions is several times higher than the workload level corresponding to VO_2_ max and this is far higher than the workload during aerobic endurance training (Laursen and Jenkins, 2002; Pescatello, 2014).

The principle that all intervals were conducted at the same TPO also meant that the perception of exertion and heart rate response tended to increase from interval to interval from a rather low level (Figure 1; also Bracken et al., 2009). This pattern provided a relatively easy entrance into the program. The participants described a consequence of this phenomenon. They felt that the first intervals were fairly easy to do, and further described that they felt tempted to put in a bit of extra effort at that point with an increase of the pedaling cadence as a consequence. However, they found that they needed to adhere to the stipulated TPO to finally have an acceptable perception of exertion at the end of the session. This result also shows that for inexperienced non-athletes, training intensity during supramaximal HIT is better regulated and controlled based on produced power (watts) and not on subjective perception of exertion or heart rate response; those variables are not constant – and instead will increase – during a session (Table 2; also Seiler and Sjursen, 2004). An apparent problem with supramaximal HIT is that consequences of overshooting the appropriate intensity during anaerobic work [e.g., lightheadedness, nausea, hypotension, syncope, and negative affective state (Lacewell et al., 2014; Sieck et al., 2016)] tend to be delayed until completion of the interval and/or the training session. Thus, if supramaximal HIT is to be successfully introduced among inexperienced older individuals, it seems advisable to control the intensity and inform participants to adhere to the stipulated TPO to avoid overshooting. This approach is what we have tried to accomplish with the program.

Another key characteristic of the program was that the intervals were conducted at a standard pedaling cadence with an individual braking force instead of a standard braking force (calculated from body weight) and free pedaling cadence as in most protocols with an all-out regimen (Bar-Or et al., 1977; Laursen and Jenkins, 2002). During the development of the program, we explored different cadence levels based on theoretical assumptions in the literature regarding acute effects associated with the relationship between power output and cadence within the predicted workload range (Jameson and Ring, 2000; Bieuzen et al., 2007; Emanuele and Denoth, 2012). We choose a pedaling cadence of 60 rpm to contribute to comfort and pleasure during warm-up, recovery, and cool-down (Zenko et al., 2016; Agricola et al., 2017). During intervals, 85 rpm was chosen based on theoretical assumptions, empirical analysis of the participants’ technical pedaling performance, and feedback from the participants in our pilot studies. This feasibility study revealed that those cadence levels seem to be applicable for persons over a large range of capability (Table 1). An interesting advantage with the standardized pedaling cadence in a group setting is that it gives an impression that everyone is exercising at the same intensity. However, a fixed braking force and free cadence mean that individuals with low capacity will have to perform the intervals at a considerably lower cadence compared to individuals with high capacity. The association suggests a disadvantage for participants with low capacity because the perception of effort at a given power output highly depends on the combination of cadence and brake force (Pandolf and Noble, 1973; Neptune and Hull, 1999; Gearhart et al., 2005; Formenti et al., 2015). The result is that it will be more difficult for participants with, for example, high body weight and low capacity to reach an adequate relative exercise intensity. In fact, emphasizing pedaling speed might not be trivial. It was recently demonstrated that a high and varying cadence *per se* seems to induce specific effects on motor control and performance among older individuals (Tsushima et al., 2015; Bellumori et al., 2017). In addition, with exercising in a group setting, free cadence carries an enhanced risk for overshooting because an individual with low capacity might be tempted to try to keep up with individuals with high capacity.

### Methodological Discussion and Limitations

First, this was a small feasibility study with the aim of developing a method using a selected group of participants and was conducted over a short training period. A longer training period is needed to explore how the methodology supports participants to further increase in training when they become ready for it, having achieved training adaptations. It should not be ignored, however, that the role of the exercise leaders regarding support and encouragement might be significant in this respect. The participants in the present study all had exercise and training experiences (e.g., resistance training and aerobic endurance training), which limits external validity. However, some of the participants had started to exercise only in recent years and had mainly experience with hydrotherapy. Of note, none of the participants had experience with watt-based cycling training, which suggests that the program might well be adapted to a general population. The participants expressed a similar perception, as revealed in the focus group interview. However, some adaptions of the program might be considered, especially if the program is to be applied for more frail older individuals. If estimation of MPO^6^ is to be derived from the BCST, the test might be further adapted with the possibility of using smaller increments (watts) than presented here (Appendix 3). To reduce the risk of overestimation of MPO^6^, one suggestion is to simply use the highest obtained power output (watts) during the BCST to derive MPO^6^ instead of the estimation described by Borg (1982). The correlation between workload at the end of escalation (watts) and highest workload (watts) during the BCST was in fact even higher (*R* = 0.97) than the correlation with the estimated MPO from the BCST (*R* = 0.91). Our data show that that the highest power output (watts) during the BCST, multiplied by 1.75, provides a usable estimate of maximum MPO^6^ (Figure A6 in Appendix 4) for prescription of training intensity. The BCST is a useful tool for estimation of maximum MPO^6^, but it is not compulsory for the use of the principles and characteristics of the program. In our pilot experiments, we also found quite good correlation between estimated maximum MPO from the BCST with other physical tests (e.g., estimated VO_2_ max and muscle strength). Thus, other options are applicable for obtaining a valid prediction of maximum capability for the duration of the interval. We chose to use the BCST because it is an externally paced anaerobic test that can be conducted with the same bike as used in the training sessions, facilitating implementation. Furthermore, among inexperienced individuals, an externally paced test such as the BCST is considered preferable compared to a self-paced test (Leone et al., 2017).

One problem with group training is that it is difficult to avoid group bias, e.g., in the rating of perceived exertion. In this study, the two leaders went up in front of each participant to individually collect ratings of perception of exertion and affective state response. However, it was not always possible to keep these individual ratings unknown to all of the other participants. Therefore, we cannot exclude the possibility that some participants were influenced by other ratings. However, judging by the intra-individual variability in ratings, we do not consider group bias to have been a significant problem.

One strength of the feasibility study is that the presented group training program was preceded by extensive pilot experiments and a series of pilot studies. We have, above and in the Appendicies, tried to describe, in as much detail as possible, how the program was developed and designed and the basis underlying related decisions. Such detailed descriptions and characteristics of studied training programs and arguments underlying the protocols are unfortunately not often offered in non-pharmacological research (Hoffmann et al., 2013; Page et al., 2017). In the present feasibility study, the presented program could easily be led by two inexperienced leaders (i.e., undergraduate students) after only a brief introduction and with passive supervision from a registered physiotherapist. This example suggests that the program has the potential to be easily implemented and applied in many contexts without the need for extensive education or experience among leaders. Another strength of the present study is the use of both quantitative and qualitative data, which allowed investigation of the feasibility criteria in a broad way and from different perspectives and dimensions. Our criteria of feasibility cover areas that are best captured using both approaches where the quantitative data and the participants’ own words complement each other.

### Implications and Conclusion

For several reasons, tailored supramaximal HIT for older individuals is of major interest for further exploration. Large, well-conducted randomized controlled trials that broadly explore both the usability and effect of supramaximal HIT among older individuals in group settings are still lacking. This study revealed that the principles and characteristics of the program seem to provide a usable methodology for further studies on supramaximal HIT among older individuals at different levels of physical capacity. The participants in this study reached a high exercise intensity in absolute and relative terms, while the methodology seems to have provided sufficient control of training intensity to ensure that the training never became too exhausting. Additionally, the training was described as pleasant to perform and was associated with positive feelings, both during and after the training sessions. Future research is needed to explore, for example, the physiological effects from, experience of, and long-term adherence to the developed supramaximal HIT program in various populations of older people in comparison with other forms of exercise training.

## Ethics Statement

This study was carried out in accordance with the recommendations of the Ethics Committee of the Medical Faculty, Umeå; University with written informed consent from all subjects. All subjects gave written informed consent in accordance with the Declaration of Helsinki. The protocol was approved by the Ethics Committee of the Medical Faculty, Umeå; University (DNR: 2016-279-31M, DNR 2016-440-32M).

## Author Contributions

ER, C-JB, MH, and NL designed the study. MH, BJ, and NL collected and analyzed the data. MH and NL drafted the manuscript. All authors interpreted data, revised the manuscript critically and approved the final version.

## Conflict of Interest Statement

The authors declare that the research was conducted in the absence of any commercial or financial relationships that could be construed as a potential conflict of interest.
