# Supplementary material for: Development and Feasibility of a Regulated, Supramaximal High-Intensity Training Program Adapted for Older Individuals
Source: Front Physiol. 2019 May 21;10:590. doi: 10.3389/fphys.2019.00590 (PMC6536694; doi:10.3389/fphys.2019.00590)
Supplement: Supplementary file 1 [file Image_1.pdf]

## Appendix 1

Establishment of the relationship between brake force level and power output.

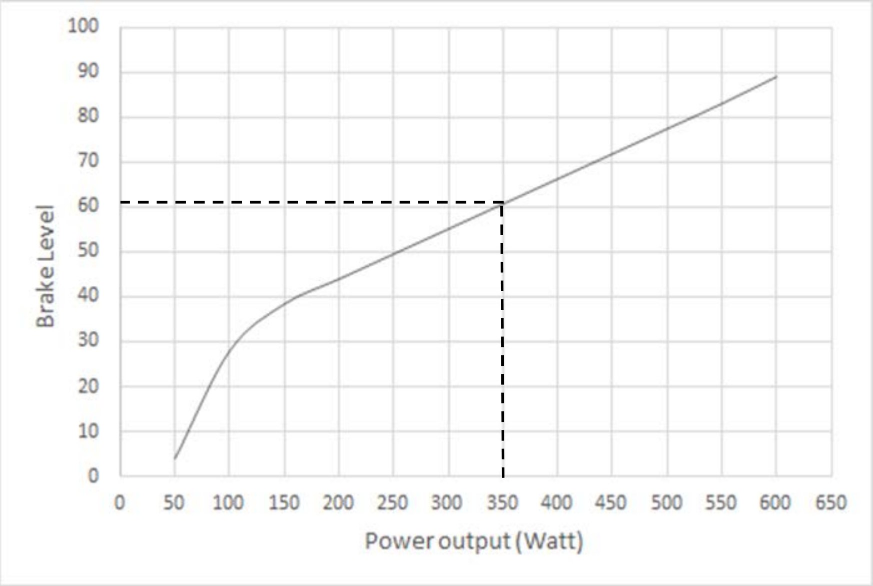


**Figure A1:** Relationship between brake force level and power output (watts) on the bike (Tomahawk IC7, Indoor Cycling Group, Nürnberg, Germany) at a pedaling cadence of 85 rpm. To establish the relationship between *Brake Level* (0-100), pedaling cadence (rpm) and Power Output (Watt), a valid and reliable power-meter (PowerTap® P1) (Pallares and Lillo-Bevia, 2018) was mounted on a bike (Tomahawk IC7, Indoor Cycling Group, Nürnberg, Germany). This allowed for continuous monitoring and synchronized sampling of heart rate, cadence and power output during testing. Required power output for each brake force level was then established for different pedaling cadences of our interest (e.g. 85 rpm). Based on the data, the brake level (0–100) for any stipulated power output (watts) could be derived by means of graphic or mathematical interpolation. The relationship was established through systematic pre-study experiments. As in the graphic example in the figure, a stipulated target power output (TPO) of 350 watts means that the brake level should be set to Level 61.

References:

Pallares, J.G., and Lillo-Bevia, J.R. (2018). Validity and Reliability of the PowerTap P1 Pedals Power Meter. *J Sports Sci Med* 17**,** 305-311.
