# Supplementary material for: Development and Feasibility of a Regulated, Supramaximal High-Intensity Training Program Adapted for Older Individuals
Source: Front Physiol. 2019 May 21;10:590. doi: 10.3389/fphys.2019.00590 (PMC6536694; doi:10.3389/fphys.2019.00590)
Supplement: Supplementary file 2 [file Image_2.pdf]

Appendix 2

Assistive utilities

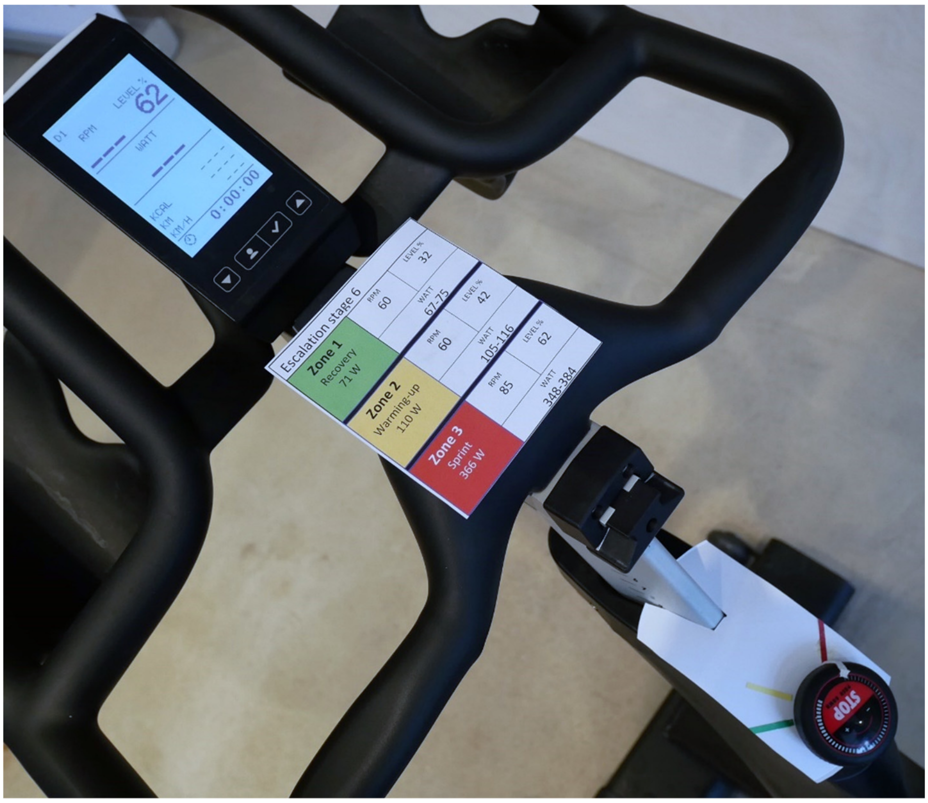

Figure A2. The picture shows the setup of assistive utilities mounted on the bike (Tomahawk IC7). In the bottom right corner of the picture, a custom-made cardboard sheet is placed under the control knob that is used to set resistance level (see figure 4 for details). In the center of the picture, there is a cardboard sheet with information about cadence and resistance level for each intensity zone (see figure 5 for details). In the top left corner, the bikes monitor is shown. The monitor provides information of actual cadence, brake level, and power output (see figure 5 for details).

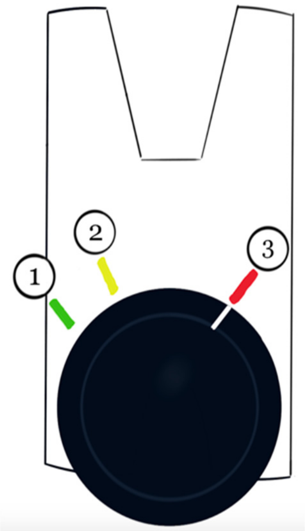

Figure A3. To make adjustment of the bike's resistance fast and easy, a custom-made cardboard sheet was attached on the bike, just under the rotatable control knob that was used to regulate the brake force level (black circle in the figure). The control knob is rotatable in 100 steps (100 levels). During training, the control knob was rotated so that the white line pointed to the correct mark on the custom-made cardboard sheet. Mark No. 1 corresponded to the individual brake force level during recovery (called *Zone 1)*. Mark No. 2 corresponded to the level during warm-up (called *Zone 2*). Mark No. 3 corresponded to the level during 6-second intervals (called *Zone 3*). The placement of the marks was determined by each individual's prescribed training load for each training session. During the training period, only the location of mark No. 3 changed. Directly after each 6-second interval, there was a pause with no resistance (i.e. the control knob was rotated counterclockwise to brake force level 0 and the pedaling cadence was free but slow.

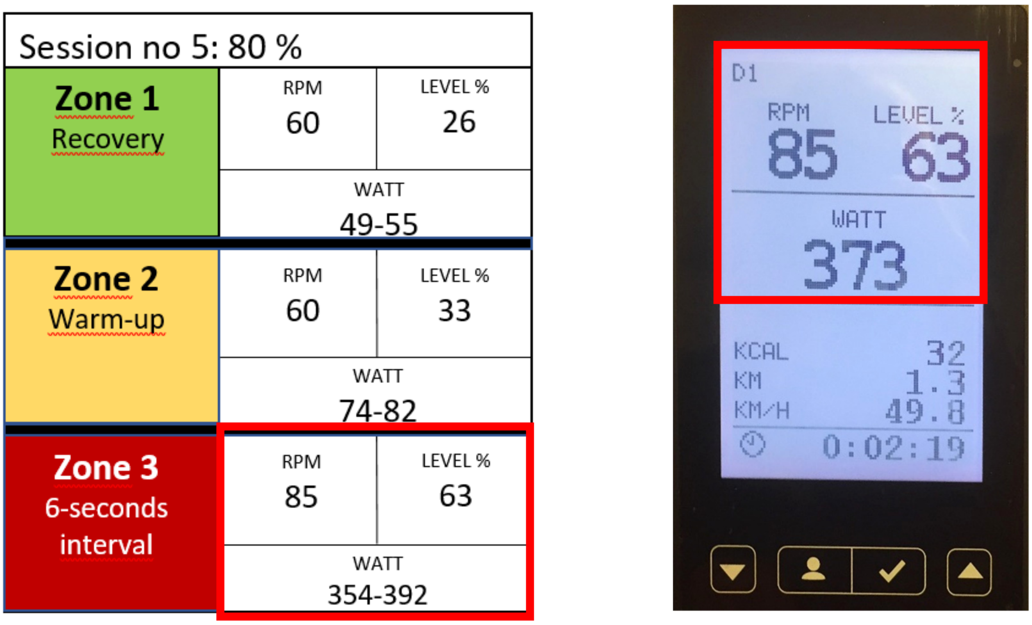

Figure A4. A cardboard sheet was attached on the handlebar (see figure 3) to provide information about stipulated pedaling cadence (RPM) and brake force level (LEVEL %) as well as the stipulated target power output (WATT ± 5%). The colors of the intensity zones were the same as the marks on the cardboard sheet presented in figure 4. The information on the cardboard was organized in the same way as the bike's monitor (highlighted by red frames).
