# Supplementary material for: Development and Feasibility of a Regulated, Supramaximal High-Intensity Training Program Adapted for Older Individuals
Source: Front Physiol. 2019 May 21;10:590. doi: 10.3389/fphys.2019.00590 (PMC6536694; doi:10.3389/fphys.2019.00590)
Supplement: Supplementary file 3 [file Image_3.pdf]

Appendix 3

Adaption and use of Borg Cycle Strength Test (BCST)

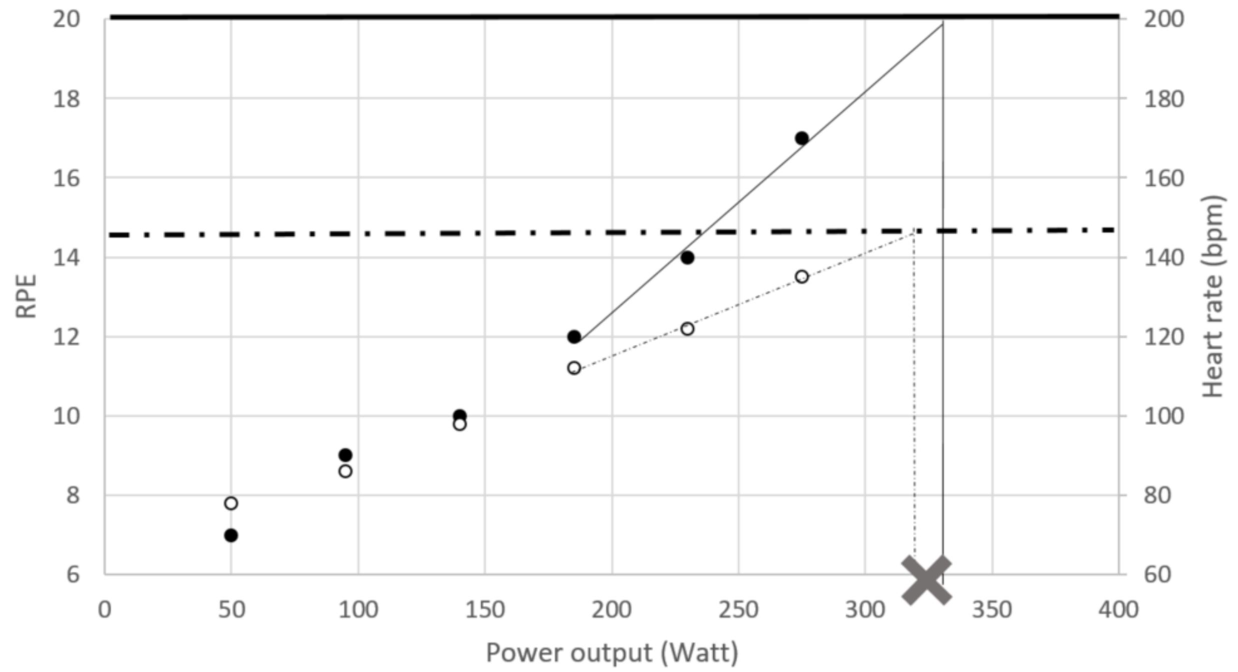

Figure A5. The figure gives a graphic description of the procedure behind estimation of maximum mean power output for a duration of 30 seconds (maximum MPO^{30}) with the Borg Cycle Strength Test (51). Rated perceived exertion and heart rate from after 30 seconds of cycling at each workload level are plotted against power output (PO). After 30 seconds of rest, workload is increased. The linear function of RPE (black dots) and PO is fitted for the three last work stages by the least-square method and extrapolated up to maximum effort (20 on the RPE scale). The function of heart rate (white dots) and PO is fitted for the three last work stages and extrapolated up to 90% of the age predicted maximum heart rate (APMHR) using the equation 208 − 0.7 × age (APMHR = 145 bpm in the example in the figure). Alternatively, if 90% of calculated APMHR was exceeded during the test, the maximum achieved heart rate during the test was used in the calculation. Estimated maximum MPO^{30} is determined by the mean of both functions. In the example in the figure, maximum MPO^{30} = 325 watts (represented by the cross on the x-axis) because the function based on RPE values was found to be 330 watts and the function based on heart rate values was found to be 320 watts. In the original BCST, described by Borg, workload started at 50 watts and increased by 50 watts for each level in the increment scheme. However, in our adapted version of the BCST, workload increased by 45 watts and 36 watts for each stage for men and women, respectively. Pedaling cadence during the adapted BCST was 85 rpm during each work stage. During rest, resistance was set to 0 and the pedaling cadence was held below 50 rpm.
