# Supplementary material for: Development and Feasibility of a Regulated, Supramaximal High-Intensity Training Program Adapted for Older Individuals
Source: Front Physiol. 2019 May 21;10:590. doi: 10.3389/fphys.2019.00590 (PMC6536694; doi:10.3389/fphys.2019.00590)
Supplement: Supplementary file 4 [file Image_4.pdf]

Appendix 4

Relationship between TPO at end of escalation and estimated maximum MPO[6].

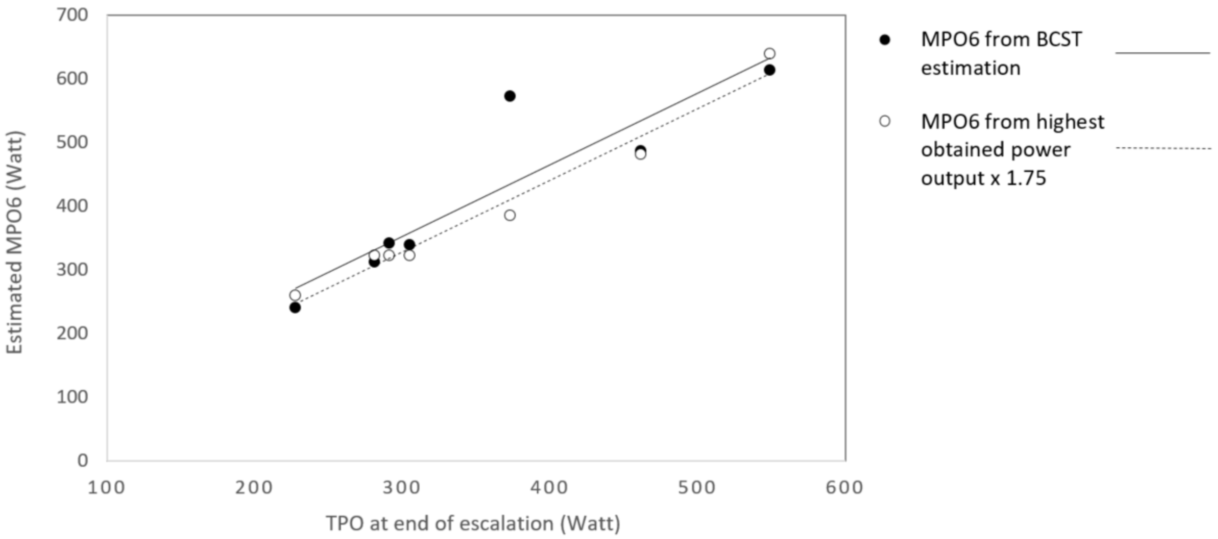

Figure A6. Black and white dots represent estimated MPO[6] from the BCST and from highest obtained power output during the BCST, respectively. The estimation from highest obtained power output is simply the power output (watts) at the last stage of the BCST × 1.75. There is a highly significant correlation between TPO at end of escalation and both methods of estimation, with high R-values (R=0.91 for estimation by the BCST and R=0.97 for estimation by highest obtained power output from BCST × 1.75). By using highest obtained power output as the basis of the estimation, the risk of overestimation of MPO[6] decreases.
